# Health-related quality of life and associated risk factors in patients with Multiple Osteochondromas: a cross-sectional study

**DOI:** 10.1007/s11136-024-03604-4

**Published:** 2024-03-08

**Authors:** Manila Boarini, Morena Tremosini, Alessia Di Cecco, Maria Gnoli, Evelise Brizola, Marina Mordenti, Elena Pedrini, Manuela Locatelli, Marcella Lanza, Diego Antonioli, Giovanni Gallone, Gino Rocca, Eric L. Staals, Giovanni Trisolino, Luca Sangiorgi

**Affiliations:** 1 Department of Rare Skeletal Disorders, IRCCS Istituto Ortopedico Rizzoli, Via di Barbiano 1/10, 40136 Bologna, Italy; 2 Unit of Pediatric Orthopedics and Traumatology, IRCCS Istituto Ortopedico Rizzoli, Bologna, Italy; 3 3rd Orthopaedic and Traumatologic Clinic Prevalently Oncologic, IRCCS Istituto Ortopedico Rizzoli, Bologna, Italy

**Keywords:** HRQoL, Skeletal dysplasia, Rare disease, Multiple Osteochondromas, PROs, Healthcare pathway

## Abstract

**Purpose:**

To evaluate the health-related quality of life and associated risk factors for Multiple Osteochondromas patients.

**Methods:**

A cross-sectional, observational study was conducted from May to December 2022 during the routine visit to the referral center for rare skeletal disorders. All patients with Multiple Osteochondromas aged ≥ 3 years were included. EuroQol 5-dimension questionnaires, and demographic, clinical, and surgical history data were collected. Descriptive statistics, Fisher’s exact test, One-sample *t*-test, Spearman’s correlation, and multiple linear and logistic regression were performed to analyze the data. Results are reported following STROBE guidelines.

**Results:**

A total of 128 patients were included in the study, with a mean age of 14 [SD, 10] years. The mean EQ-5D Index Value was 0.863 [SD, 0.200] and the EQ-VAS was 84 [SD, 19] with a positive correlation between two scores [*r* = 0.541, *p* < 0.001]. Patients frequently referred problems in pain/discomfort [78.8%], anxiety/depression [50%], and usual activities [38.8%] dimensions. Increasing age was the common risk factor for health-related quality of life [*p* < 0.000], as well as Index Value and VAS scores were significantly lower in surgical patients [*p* = 0.001 and *p* < 0.001, respectively].

**Conclusion:**

Increasing age and surgical procedures were found highly associated with reduced health-related quality of life in Multiple Osteochondromas patients. Our findings provide relevant information to support the establishment of patient-centered healthcare pathways and pave the way for further research into medical and non-medical therapeutic strategies for these patients.

**Supplementary Information:**

The online version contains supplementary material available at 10.1007/s11136-024-03604-4.

## Plain English Summary

Multiple Osteochondromas (MO) is a lifelong rare genetic and chronic condition presenting in childhood. However, there is limited research on the Health-related quality of life (HRQoL) of patients with MO. In a real-world clinical setting, we found that using the EuroQol 5-dimensions (EQ-5D) questionnaires can help identify risk factors for HRQoL in MO patients. It underlines the negative impact of increasing age and surgical procedures from eight years onward. Meaningful worse HRQoL scores were found across several domains of the EQ-5D questionnaire, in particular pain/discomfort, anxiety/depression, and usual activities. Multiple Osteochondromas is a rare skeletal disease with a chronic course. Therefore, the focus of care should be on prevention and early detection of late complications, beginning in childhood. Our findings integrate the patient experience and clinical examination, providing a comprehensive understanding of patient needs. It promotes the creation and development of effective, dedicated, patient-centered, and multidisciplinary healthcare pathways.

## Background

There are approximately 7000 distinct rare diseases (RDs), a wide variety of conditions that affect a relatively small amount of the world’s population [1–3]. RDs are a multifaceted, chronic, and life-threatening group of disorders, leading to common social and healthcare challenges that are frequently associated with physical limitations, psychological distress, and a lower quality of life (QoL) [4–8].

Multiple Osteochondromas (MO—MIM, #133700 and #133701) is an inherited autosomal dominant genetic disorder marked by osteochondromas (OCs), osteocartilaginous outgrowths [9, 10]. The number and size of OCs increase during childhood and remain stable after the closure of the growth plate [11]. The disruption of osseous growth, long bone bowing, angulation of the joints, and a reduction in range of motion are the most prevalent clinical concerns related to MO [12–16]. Malignant transformation of an existing OC into secondary peripheral chondrosarcoma during adulthood is the most feared complication, reported in about 5% of patients [9].

Health-related quality of life (HRQoL) is the assessment of how satisfied an individual is with their overall health and well-being, considering that the person is the primary source of information about their health [17]. Previous studies have found that the MO population has a reduced HRQoL compared to the general population due to clinical manifestations, impacting daily activities as well as social and psychological well-being [18–23].

Since HRQoL is a subjective measure, one of the best ways to assess it is through questionnaires or patient-reported outcome measurements (PROMs) [24, 25]. The EQ-5D^®^ is the world’s most widely used tool [26–28].

The EQ-5D^®^ is a set of generic, multi-dimensional, available for different age ranges of users, and utility tools. Due to their ease of applicability, are effective for a variety of conditions and suitable for different purposes, from routine clinical settings to clinical trials as well as for medical decision-making [29–32].

Further studies regarding assessment, management, and care for MO’s HRQoL are needed, including cost-utility analyses as already highlighted by several international health technology assessment (HTA) agencies [33].

Thus, this study aimed to evaluate HRQoL in a large cohort of MO individuals and to investigate its potential risk factors.

## Methods

### Study design, setting, and ethical statement

This research is part of an ongoing prospective study on MO, the Registry of Multiple Osteochondromas (REM—NCT04133285) [34]. We conducted a cross-sectional investigation of HRQoL in MO patients between May and December 2022 at the rare skeletal disorders outpatient clinic (CeMaRS) of the IRCCS Istituto Ortopedico Rizzoli in Bologna, Italy.

The CeMaRS is a center with a multidisciplinary approach dedicated to the diagnosis and treatment of rare skeletal disorders, including MO. During the daily clinical routine, patients receive a multidisciplinary examination tailored to their needs, including genetic counseling, orthopedic and rehabilitation visits, radiological investigations, and psychological support. Since May 2022, the EQ-5D questionnaire has been gradually introduced into clinical routine to recognize factors impacting the HRQoL of these individuals.

The Strengthening Reporting of Observational Studies in Epidemiology (STROBE) checklist for cross-sectional study was adopted in reporting this research (S1) [35].

The study was approved by the Local Ethical Committee (protocol number 0021283/2013) and conducted in accordance with the 1964 Declaration of Helsinki. Informed consent was obtained from each adult participant and the parents/guardians of participants under 18-years-old.

### Participants

The study population consisted of a series of consecutive patients under treatment in the CeMaRS. The subjects were screened consecutively and considered eligible when met the following inclusion criteria: (a) age ≥ 3 years old; (b) clinical diagnosis of MO; (c) Italian native language; (d) complete clinical data available; and (e) enrolled in the Registry of Multiple Osteochondromas. The individuals were excluded from the analysis due to the following criteria: (a) age less than 3 years; (b) established diagnosis of a distinct, rare skeletal disease; (c) healthy patients; (d) undergoing evaluation for differential diagnosis; (e) declination to provide consent for REM; and (f) incomplete data.

### Measures

#### Demographic, clinical, and surgical measures

Demographic variables included sex at birth and age at visit (in years). Height, weight, and body mass index (BMI) were calculated as absolute values as well as *z*-scores. The *z*-score was calculated using Italian growth charts [36].

**Fig. 1:**
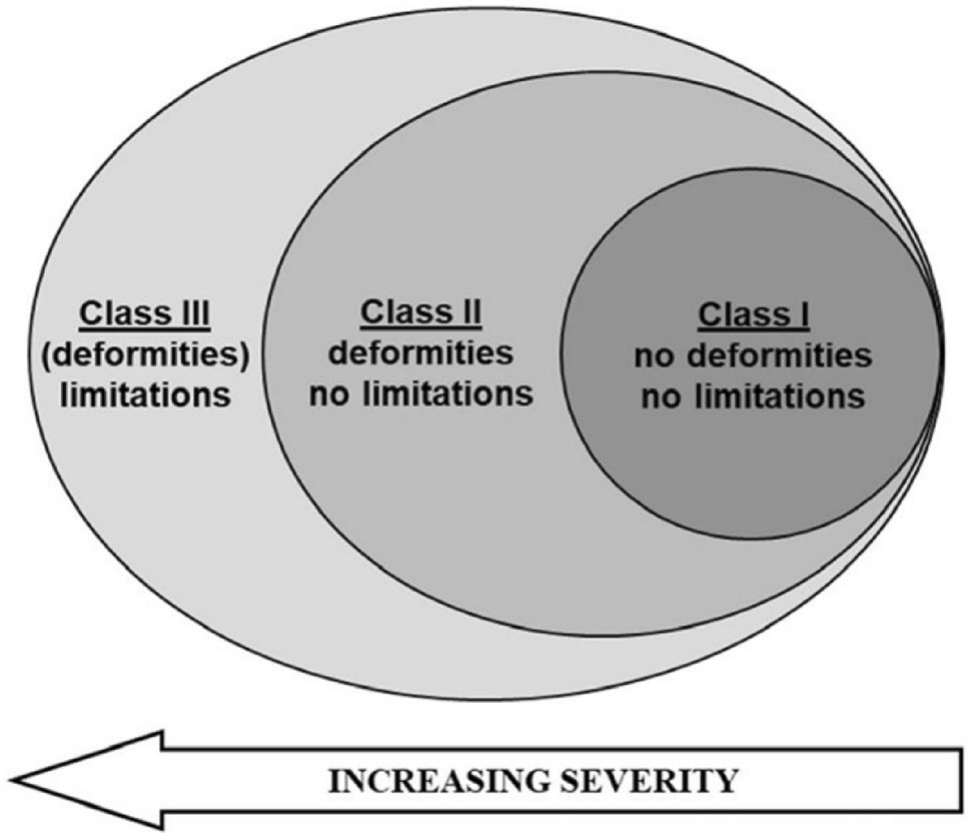
The Rizzoli multiple osteochondromas classification. A visual representation of the three MO classes [37]

The following MO-related clinical features were routinely collected during the visits by experienced pediatric and adult orthopedic surgeons: number and locations of OCs; deformities refer to skeletal deviations from normal shape, size, or alignment, including genu valgum/varum, leg-length discrepancy, and forearm procurvation; functional limitations are alterations from the normal range of motion; and disease severity, assessed by the revised IOR MO Classification. This classification system groups MO severity into three classes (I, II, and III) based on phenotype manifestation refers to the presence/absence of osteochondromas, skeletal deformities, and functional limitations; severity increases from class I to class III (Fig. 1) [11, 37]. In addition, the age at the first surgery and the total number of surgical procedures were collected from patients’ health medical records.

#### Outcome measures

Allied professionals assessed HRQoL using the validated Italian version of the EQ-5D tools: (a) EQ-5D-5L for adults ≥ 16 years; (b) EQ-5D-Y for adolescents between 8 and 15 years; and (c) EQ-5D-Y Parent-Proxy for children < 8 years [38, 39].

The questionnaire consists of two sections—descriptive system and visual analog scale (VAS)—and yields three scores: health profile, Index Value (IV), and overall health status (VAS) [40].

In the descriptive system section, patients report their level of problems in five dimensions—mobility, self-care, usual activities, pain/discomfort, and anxiety/depression—and rate their health on a 5-level options for adults, and 3-level for adolescents and children. Based on the pattern of their responses, patients generate a health profile ranging from best health (11111 = highest level for adults and adolescents/children) to worst health (55555 = lowest level for adults; 33333 = lowest level for adolescents/children). Each health profile is transformed into an IV, a summary index that represents the individual’s perception of their own health within country-specific preferences. The index varies from 1 (perfect health) to 0 (absence of life/death), with values less than zero reflecting health states “worse than death”.

The EQ-VAS measures the patients’ overall health status, with a score ranging from 0 (“The worst health you can imagine”) to 100 (“The best health you can imagine”).

### Data collection

Data collection comprised the following information: the anthropometric evaluations were measured using standardized techniques; the presence, number, and location of clinical features and IOR MO class were assessed through comprehensive physical examinations and supported by imaging (i.e., radiography, magnetic resonance imaging). Lastly, the EQ-5D questionnaire was administered to measure health-related quality of life.

Each scheduled appointment at CeMaRS included data collection, which was subsequently captured in the REM. No additional exams or investigation were required; only information gathered during routine examinations was recorded within REM.

REM is a standardized disease registry that stores clinical, genetic, and family history as well as quality of life data from patients with MO. It is reachable via a web-accessible platform and is in compliance with national and European privacy regulations and medical informatics standards.

### Statistical analysis

Descriptive statistics were provided by analyzing patients’ characteristics and EQ-5D scores. Continuous variables were summarized using the mean, standard deviation [SD], median, and interquartile range [IQR]. Categorical variables were reported as counts and percentages. Fisher’s exact test was used for non-metric variables. One-sample *t*-tests were used to compare the IV and VAS scores of MO adult patients to Italian population norms. Spearman’s rank correlation coefficient was used to calculate correlations between IV and VAS.

The main outcome was HRQoL, measured by the EQ-5D health profile, IV, and VAS. EQ-5D IV was obtained by applying preference weights from the health value scale defined according to a 5-level EQ-5D scale for the Italian population, ranging from − 0.573 (worst health) to 1.000 (best health) for adults [41]. Due to the unavailability of the Italian value set for the pediatric population, the Spanish EQ-5D-Y value set was used as a reference in this study, as per EuroQol recommendations [42, 43]. In addition, for all five dimensions, all the levels ≥ 2 were grouped and thus dichotomized to “no problem” or “any problem”, according to EuroQol Group guidelines [41].

The linear regression model was used to identify associations between EQ-VAS, EQ-5D IV, and patient characteristics; results were presented as mean difference [MD] with 95% confidence intervals [CI] and *p*-value. The Five Dimensions were analyzed using the logistic regression model to identify associations between domains and patient characteristics; results were presented as odds ratio [OR] with 95%CI and *p*-value. To assess the impact of potential confounding factors (disease-related features), we constructed three multivariable models and added the confounding factors sequentially. Multivariable models were estimated by performing multiple imputations to account for missing data in the predictors that were evaluated. Combined estimates were obtained from 20 imputed data sets.

The statistical analyses were performed using SPSS v. 25.0 (SPSS, Chicago, IL, USA) and STATA v.11.2 (STATA Corp., College Station, TX, USA).

## Results

### MO patients’ characteristics

**Fig. 2:**
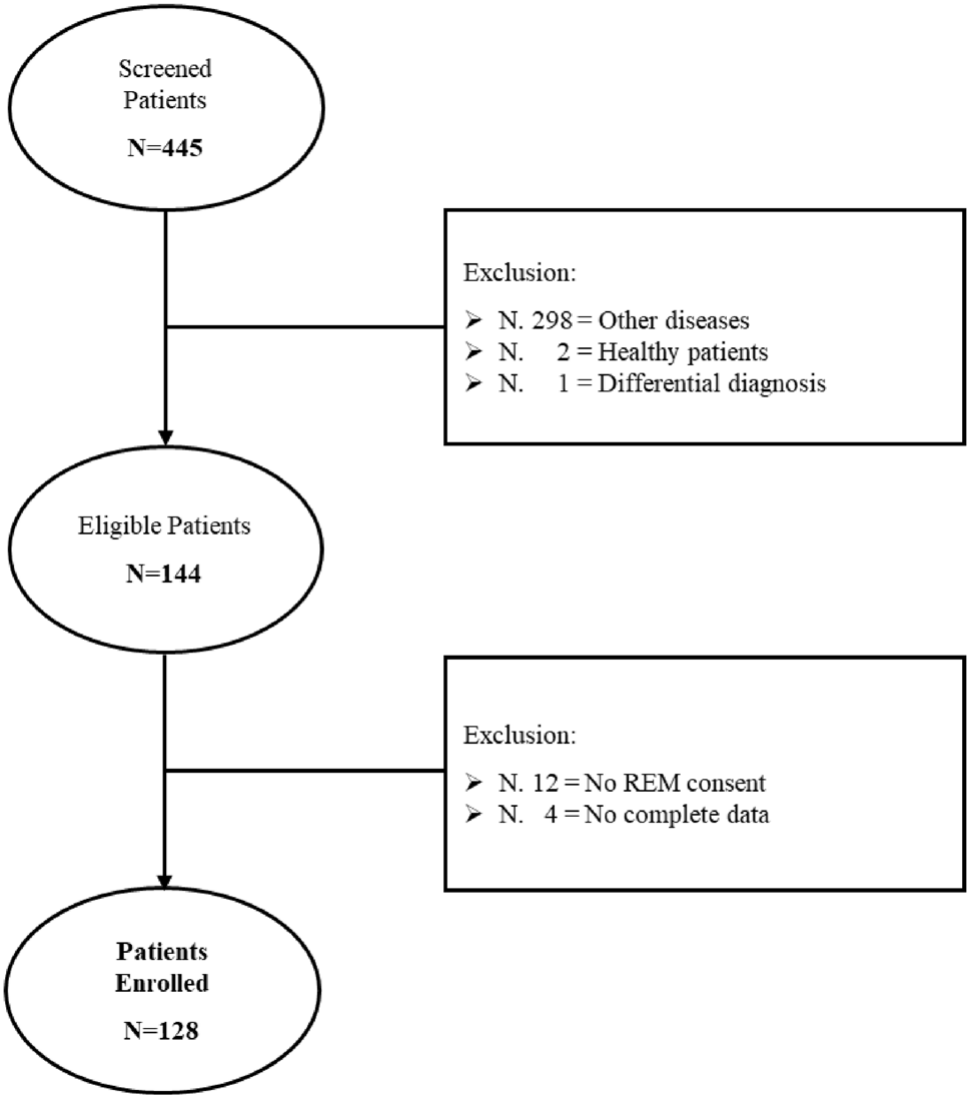
Flowchart of MO patients included in the study

A total of 445 patients were treated at CeMaRS, from May to December 2022, of whom 128 met the study inclusion criteria (Fig. 2).

Overall, the mean age was 14 years [SD, 10 years], accounting for 52.3% of females.

Patients with MO had slightly lower height, weight, and BMI z-scores than the Italian population [− 0.87 [SD, 0.82], − 0.28 [SD, 1.27], and − 0.10 [SD, 1.17], respectively]. The median number of OCs was ten [IQR, 0–35] with 54.7% of patients presenting ten or more osteochondromas. The median number of deformities was two [IQR, 0–11] with 53.9% exhibiting two or more deformities. The median number of functional limitations was zero [IQR, 0–5] with 27.3% manifesting one or more limitations. Most patients were classified within the IOR Class II [45.4%]; the other ones were equally distributed in Class I and III [27.3% for each class]. Furthermore, 42.2% of patients underwent surgical procedures, with a median number of two [IQR, 1–7] and a mean age at first treatment of 11 years [SD, 7 years]. Detailed MO patients’ characteristics are reported in Table 1.

**Table 1:** Demographic, clinical, and surgical characteristics of MO patients

Characteristics		Overall (*N* = 128)
Demographic features		
Age, years	Mean [SD]	14 [10]
Sex	*N* [%]	
Male		61 [47.7%]
Female		67 [52.3%]
Comorbidity	*N* [%]	
No		113 [88.3%]
Yes		15 [11.7%]
Anthropometric features		
Height^a^	Mean [SD]	
cm		158.50 [10.62]
*z*-score		− 0.87 [0.82]
Weight^b^	Mean [SD]	
kg		40 [17.67]
*z*-score		− 0.28 [1.27]
BMI^b^	Mean [SD]	
kg/m^2^		18.18 [4.90]
*z*-score		− 0.10 [1.17]
Clinical features		
IOR classification	*N* [%]	
Class I		35 [27.3]
Class II		58 [45.4]
Class III		35 [27.3]
N. of OCs	Median [IQR]	10 [0, 35]
Upper limbs OCs		4 [0–26]
Lower limbs OCs		5 [0–18]
Trunk OCs		0 [0–4]
N. of deformities	Median [IQR]	2 [0–11]
Upper limbs deformities		0 [0–6]
Lower limbs deformities		1 [0–10]
Trunk deformities		0 [0–3]
N. of limitations	Median [IQR]	0 [0–5]
Upper limbs limitations		0 [0–4]
Lower limbs limitations		0 [0–4]
Trunk limitations		0 [0–1]
Surgery	*N* [%]	
No		74 [57.8%]
Yes		54 [42.2%]
Age at first surgery, years	Mean [SD]	11 [7]
N. of surgeries	Median [IQR]	2 [1–7]

*SD* standard deviation; *IQR* interquartile; *BMI* body mass index; *OCs* osteochondromas

^a^Data were missing for 13 (10.2%) patients

^b^Data were missing for 17 (13.3%) patients

The comparison between the age groups (children, adolescents, and adults) confirmed that the phenotypic presentation worsened with age [*p* < 0.001], number of OCs [*p* = 0.001], and number of deformities [*p* < 0.001] (Supplementary Table 1). Significant differences were noted between patients who underwent surgery versus those who did not [*p* < 0.001]. Surgical patients exhibited a higher number of OCs, deformities, and functional limitations [*p* < 0.001, *p* = 0.02, and *p* = 0.04, respectively]. Furthermore, they were significantly associated with the IOR Class III [*p* = 0.005] (Supplementary Table 2). Lastly, no significant sex-related differences were observed (Supplementary Table 3).

### MO patients’ HRQoL description

Table 2 provides a detailed MO HRQoL description. The mean EQ-5D IV was 0.863 [SD, 0.200], ranging from 0.934 to 0.735. The mean EQ-VAS score was 84 [SD, 19], ranging from 97 to 67.

**Table 2:** MO patient HRQoL description

HRQoL	< 8 years (*N* = 29)	8–15 years (*N* = 68)	≥ 16 years (*N* = 31)	Overall (*N* = 128)
Mobility				
No problems	25 [86.2%]	59 [86.8%]	16 [51.6%]	100 [78.1%]
Any problems^a^	4 [13.8%]	9 [13.2%]	15 [48.4%]	28 [21.9%]
Self-care				
No problems	29 [100%]	64 [94.1%]	21 [67.7%]	114 [89.1%]
Any problems	0 [0%]	4 [5.9%]	10 [32.3%]	14 [10.9%]
Usual activities				
No problems	27 [93.1%]	56 [82.4%]	14 [45.2%]	97 [75.8%]
Any problems	2 [6.9%]	12 [17.6%]	17 [54.8%]	31 [24.2%]
Pain/discomfort				
No problems	22 [75.9%]	36 [52.9%]	7 [22.6%]	65 [50.8%]
Any problems	7 [24.1%]	32 [47.1%]	24 [77.4%]	63 [49.2%]
Anxiety/depression				
No problems	28 [96.6%]	46 [67.6%]	14 [45.2%]	88 [68.7%]
Any problems	1 [3.4%]	22 [32.4%]	17 [54.8%]	40 [31.3%]
Any dimension				
No problems	19 [65.5%]	26 [38.2%]	3 [9.7%]	48 [37.5%]
Any problems	10 [34.5%]	42 [61.8%]	28 [90.3%]	80 [62.5%]
EQ-5D Index Value	0.934 [0.116]	0.890 [0.110]	0.735 [0.326]	0.863 [0.200]
EQ-VAS	97 [6]	86 [14]	67 [25]	84 [19]

Data are expressed as *N* [%] and mean ± standard deviation [SD]

^a^All levels ≥ 2 was collapsed

Overall, 62.5% of the patients reported “any problems” in at least one of the EQ-5D dimensions, and pain/discomfort was the most frequent of them [78.8%], followed by anxiety/depression [50%] and usual activities [38.8%], whereas problems in the dimension of self-care were the least frequent [17.5%]. In detail, adults and adolescents indicated more frequently “any problems” regarding pain/discomforts [77.4% and 47.1%, respectively], anxiety/depression [54.8% and 32.4%, respectively], and usual activities [54.8% and 17.6%, respectively] than parent-proxy reported about their children [24.1%, 3.4%, and 6.9%, respectively] (Supplementary Figure 1).

### EQ-5D stratified by age, sex, and surgical procedures

EQ-5D IV was significantly associated with age and surgical procedure. The adults reported lower IV than the adolescent and children’s groups [1.000 vs 0.887 vs 0.859, *p* < 0.001], and those who underwent surgery reported poorer IV scores than those who did not [0.955 vs 0.861, *p* = 0.001]. Likewise, the adult group and surgical patients reported a significantly worse EQ-VAS score [*p* < 0.001 for both].

Considering the five EQ-5D dimensions, the adults referred significantly lower scores for mobility, self-care, usual activities, pain/discomfort, and anxiety/depression [*p* < 0.001 for all dimensions], and surgical patients exhibited diminished scores in each dimension [*p* = 0.025, *p* = 0.019, *p* = 0.040, *p* = 0.022, *p* = 0.002, respectively].

No differences were identified between the sexes. Table 3 summarizes the findings.

**Table 3:** EQ-5D stratified by age groups, sex, and surgery (*N* = 128)

EQ-5D health score	Age groups				Sex			Surgery		
	< 8 years (*N* = 29)	8–15 years (*N* = 68)	≥ 16 years (*N* = 31)	*p* value	Male (*N* = 61)	Female (*N* = 67)	*p* value	No (*N* = 74)	Yes (*N* = 54)	*p* value
EQ-5D Index Value										
Median	1.000	0.887	0.859	< 0.001	0.914	0.887	0.93	0.955	0.861	0.001
IQR^a^	0.887, 1	0.825, 1	0.613, 0.953		0.811, 1	0.825, 1		0.825, 1	0.781, 0.955	
EQ-5D VAS scale										
Median	100	90	75	< 0.001	85	95	0.42	96	80	< 0.001
IQR	95, 100	79, 100	50, 85		80, 100	75, 100		86, 100	70, 90	
Five dimensions										
Mobility										
No problems	25 [86.2%]	59 [86.8%]	16 [51.6%]	< 0.001	46 [75.4%]	54 [80.6%]	0.48	63 [85.1%]	37 [68.5%]	0.025
Any problems^a^	4 [13.8%]	9 [13.2%]	15 [48.4%]		15 [24.6%]	13 [19.4%]		11 [14.9%]	17 [31.5%]	
Self-care										
No problems	29 [100%]	64 [94.1%]	21 [67.7%]	< 0.001	54 [88.5%]	60 [89.6%]	0.85	70 [94.6%]	44 [81.5%]	0.019
Any problems	0 [0%]	4 [5.9%]	10 [32.3%]		7 [11.5%]	7 [10.4%]		4 [5.4%]	10 [18.5%]	
Usual activities										
No problems	27 [93.1%]	56 [82.4%]	14 [45.2%]	< 0.001	43 [70.5%]	54 [80.6%]	0.18	61 [82.4%]	36 [66.7%]	0.040
Any problems	2 [6.9%]	12 [17.6%]	17 [54.8%]		18 [29.5%]	13 [19.4%]		13 [17.6%]	18 [33.3]	
Pain/discomfort										
No problems	22 [75.9%]	36 [52.9%]	7 [22.6%]	< 0.001	33 [54.1%]	32 [47.8%]	0.47	44 [59.5%]	21 [38.9%]	0.022
Any problems	7 [24.1%]	32 [47.1%]	24 [77.4%]		28 [45.9%]	35 [52.2%]		30 [40.5%]	33 [61.1%]	
Anxiety/depression										
No problems	28 [96.6%]	46 [67.6%]	14 [45.2%]	< 0.001	46 [75.4%]	42 [62.7%]	0.12	59 [79.7%]	29 [53.7%]	0.002
Any problems	1 [3.4%]	22 [32.4%]	17 [54.8%]		15 [24.6%]	25 [37.3%]		15 [20.3%]	25 [46.3%]	

Data are expressed as N [%]

*IQR* interquartile

^a^All levels ≥ 2 were collapsed

### Correlation between EQ-5D Index Value and EQ-VAS and comparison with Italian population norms

**Fig. 3:**
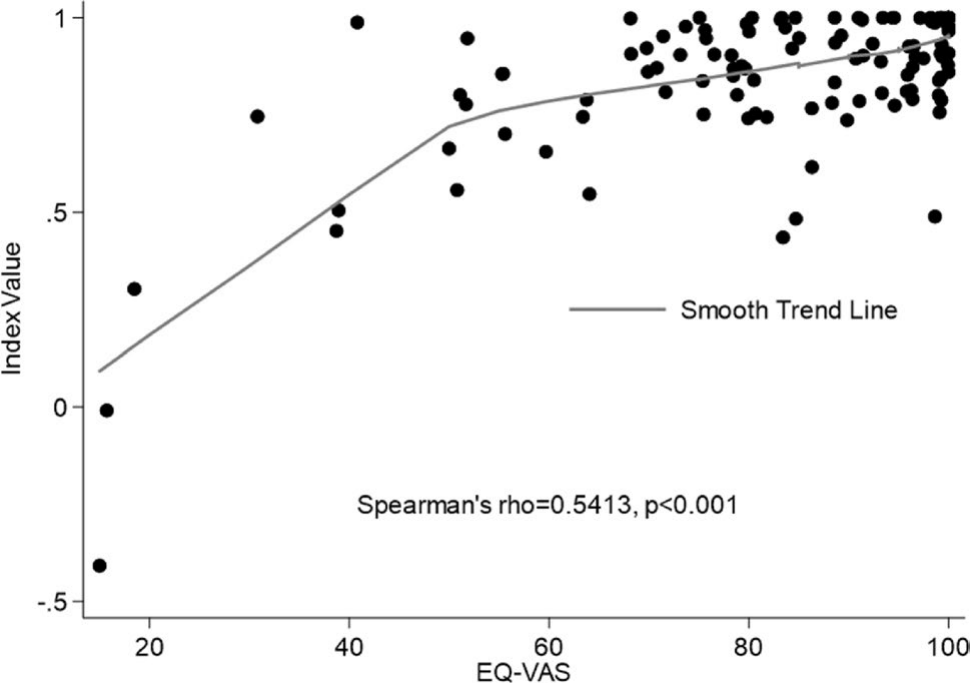
Correlation between EQ-5D Index Value and EQ-VAS

A moderate positive correlation between IV and VAS scores was observed, as displayed in Fig. 3 [*ρ* = 0.541, *p* < 0.001].

We performed One-sample t-test to compare the IV and VAS scores of MO adult patients with the Italian norms. MO patients reported significantly lower IV [*M* = 0.71, *SD* = 0.34] and VAS [*M* = 66, *SD* = 26.9] than the adult Italian population [*t*(25) = − 3.32, *p* = 0.003 and *t*(25) = − 3.01, *p* = 0.006, respectively].

### Multiple linear and logistic regression analyses on EQ-5D

Univariate analyses were performed to investigate the effects of each factor on the IV, EQ-VAS, and Five Dimensions.

Increasing age and comorbidities were associated with a worse IV [*p* < 0.0005 and *p* = 0.024, respectively]. MO clinical features associated with significantly lower IV scores were: trunk OCs [*p* = 0.029], increasing number of deformities and functional limitations [*p* = 0.004 and *p* = 0.037, respectively], and previous surgeries [at least one, *p* = 0.003 and two or more, *p* = 0.036, respectively].

Rising age, trunk deformities, and at least one/two or more previous surgeries were associated with a worse VAS score [*p* < 0.000, *p* = 0.007, *p* = 0.002, and *p* < 0.0005, respectively].

The Five Dimensions were negatively associated with increasing age [*p* < 0.0005, *p* = 0.005, *p* < 0.0005, *p* = 0.001, and *p* = 0.001]. Patients with at least one surgery showed a worse score in mobility, self-care, and anxiety/depression dimensions [*p* = 0.044, *p* = 0.007, and *p* = 0.018, respectively]. The self-care score decreased significantly with an increasing number of trunk OCs [*p* = 0.008], deformities [*p* = 0.016], and lower limb functional limitations [*p* = 0.046]. Usual activities were negatively affected by the increasing number of lower limb OCs and two or more surgical procedures [*p* = 0.007 and *p* = 0.043, respectively]. The pain/discomfort dimension was negatively associated with an increasing number of trunk OCs and two or more surgical procedures [*p* = 0.005 and *p* = 0.049, respectively]. The increasing number of lower limb OCs, functional limitations, and undergoing two or more surgeries determined a significantly worse anxiety/depression state [*p* = 0.009, *p* = 0.023, and *p* = 0.007, respectively].

Multiple linear and logistic regression models were performed to investigate the effects of combining factors on IV, VAS, and the Five Dimensions.

EQ-5D IV was negatively associated with increasing age, increasing number of OCs and deformities located in the trunk [*p* < 0.0005, *p* = 0.006, and *p* = 0.010, respectively] in Model 1; with increasing age, comorbidities, and number of deformities [*p* < 0.0005, *p* = 0.027, and *p* = 0.029, respectively] in Model 2; and lastly, with increasing age and comorbidities [*p* < 0.0005 and *p* = 0.017, respectively] in Model 3.

EQ-VAS score was negatively associated with increasing age, increasing number of OCs and functional limitations located in the trunk, and increasing number of lower limb deformities [*p* < 0.0005, *p* = 0.005, *p* = 0.039, and *p* = 0.048, respectively] in Model 1; with increasing age and two or more surgical procedures [*p* < 0.0005 and *p* = 0.007, respectively] in Model 2; and finally, with increasing age and at least one/two or more surgeries [*p* < 0.0005, *p* = 0.033 and *p* = 0.002, respectively] in Model 3.

Overall, increasing age was the most common risk factor for each dimension in all three models [*p* < 0.000]. Nonetheless, other factors negatively impacted the dimensions. In detail, self-care problems were influenced by comorbidities [Model 1: *p* = 0.050; Model 2: *p* = 0.021; Model 3: *p* = 0.025], increasing number of trunk OCs [*p* = 0.013] in Model 1, and by at least one surgery [*p* = 0.037] in Model 3. Usual activities problems were negatively conditioned by an increasing number of lower limb OCs [*p* = 0.006], whereas pain/discomfort complaints were impacted by an increasing number of trunk OCs [*p* = 0.003] in Model 1.

All the results are presented in Tables 4 and Supplementary Table 4.

**Table 4:** Multiple linear and logistic regression analyses on EQ-5D Index Value (*N* = 128)

Characteristics	EQ-5D Index Value											
	Univariate regression			Multiple regression								
	MD	95% CI	*p*	Model 1^a^			Model 2^b^			Model 3^c^		
				MD	95% CI	*p*	MD	95% CI	*p*	MD	95% CI	*p*
Age at visit, *per* 1-year increase	− 0.01	− 0.01, − 0.01	0.000	− 0.01	− 0.01, − 0.01	0.000	− 0.01	− 0.01, − 0.01	0.000	− 0.01	− 0.01, − 0.01	0.000
Comorbidity												
Without (ref.)												
With	− 0.12	− 0.23, − 0.02	0.024	− 0.08	− 0.17, 0.01	0.089	− 0.10	− 0.20, − 0.01	0.027	− 0.11	− 0.21, − 0.02	0.017
BMI *z*-score, per 1-point increase	− 0.01	− 0.04, 0.01	0.385	0.00	− 0.02, 0.02	0.875	0.01	− 0.02, 0.03	0.576	0.00	− 0.02, 0.03	0.914
N. of OCs, per 1-OC increase	− 0.00	− 0.01, 0.00	0.202				0.00	− 0.00, 0.01	0.462			
ULs OCs	− 0.00	− 0.01, 0.01	0.744	0.01	− 0.00, 0.01	0.258						
LLs OCs	− 0.01	− 0.01, 0.00	0.237	0.00	− 0.01, 0.01	0.469						
Trunk OCs	− 0.04	− 0.08, − 0.00	0.029	− 0.04	− 0.07, − 0.01	0.006						
N. of Def per 1-Def increase	− 0.02	− 0.03, − 0.01	0.004				− 0.02	− 0.03, − 0.00	0.029			
ULs Def	− 0.04	− 0.06, − 0.02	0.001	− 0.02	− 0.05, 0.00	0.086						
LLs Def	− 0.01	− 0.03, 0.02	0.595	0.00	− 0.02, 0.02	0.657						
Trunk Def	− 0.13	− 0.20, − 0.06	0.000	− 0.09	− 0.16, − 0.02	0.010						
N. of Lim per 1-Lim increase	− 0.04	− 0.07, − 0.00	0.037				− 0.01	− 0.05, 0.02	0.449			
ULs Lim	− 0.02	− 0.07, 0.03	0.467	− 0.02	− 0.07, 0.04	0.526						
LLs Lim	− 0.07	− 0.12, − 0.01	0.014	− 0.05	− 0.10, 0.00	0.070						
Trunk Lim	0.03	− 0.37, 0.42	0.883	0.16	− 0.23, 0.56	0.409						
IOR class												
Class I (ref.)												
Class II	− 0.04	− 0.12, 0.05	0.385							− 0.00	− 0.08, 0.08	0.985
Class III	− 0.07	− 0.16, 0.03	0.167							− 0.02	− 0.12, 0.07	0.590
Surgery												
Without (ref.)												
At least 1 surgery	− 0.13	− 0.21, − 0.04	0.003	− 0.05	− 0.14, 0.03	0.207	− 0.06	− 0.15, 0.02	0.143	− 0.08	− 0.16, 0.01	0.081
≥ 2 surgeries	− 0.09	− 0.18, − 0.01	0.036	− 0.03	− 0.12, 0.05	0.446	− 0.05	− 0.14, 0.03	0.231	− 0.05	− 0.13, 0.03	0.265

*MD* mean difference; *CI* confidence interval; *OCs* osteochondromas; *ULs* upper limbs; *LLs* lower limbs; *Def* deformities; *Lim* limitations

^a^Model 1: adjusted by all the variables in the Model 1

^b^Model 2: adjusted by all the variables in the Model 2

^c^Model 3: adjusted by all the variables in the Model 3

## Discussion

MO is a rare and chronic skeletal condition that negatively impacts QoL and requires long-term health care [18, 19].

The key findings of our study are that increasing age and the number of surgeries harmed MO patients’ HRQoL.

Consistently with previous studies, our data confirm the lifespan persistence of disease-related problems with a significant worsening of the health status in adults [18–20, 22].

Interestingly, participants who responded for themselves (adolescents and adults) reported different perceptions from proxy responders, parents for children. In our cohort, parents reported significantly better overall quality of life and in each dimension than the two other groups. Three of the six previous studies that investigated MO patients’ quality of life reported opposing results. Our findings are consistent with Chinna et al., the authors concluded that parents who were affected by MO and answered the survey reported better QoL since, due to their own experience, they learned to adapt to the condition [19]. Caino et al. reported no significant differences due to the limited sample size [22]. A plausible explanation is that parents developed the capacity to identify and cope with disease-related issues over time, providing their children with safety and quiet [6]. Conversely, Sundin Palmeira de Oliveira et al. showed that parents’ perceptions were worse than patients’ ones, suggesting that parents’ health and well-being may influence on how well they rate their child’s well-being [23]. Nevertheless, the difference in perception between parents and children remains a sensitive and poorly investigated problem that should be further explored and included in the decision-making process, especially when corrective surgery is proposed.

Overall, the univariate analyses confirmed the negative effects of increasing age and number of surgeries on IV, EQ-VAS score, and five dimensions, and all three multiple regression models validated their significant impact. Our results are consistent with the unique previous Italian study [20] showing that HRQoL decreases as the number of surgeries increases. Nevertheless, surgery remains the treatment of choice for patients with MO. This means that patients may undergo multiple surgeries over the course of their lives, especially during childhood and adolescence. The impact of repeated surgeries during developmental age has been described in other diseases. Rang used the term “birthday syndrome” to describe the situation where children with cerebral palsy (CP) would undergo surgery every year. To overcome this issue, novel strategies, such as single-event multilevel surgery, have been developed in CP, aiming at optimizing clinical and functional results while minimizing the need of recurrent surgical interventions [44]. We hypothesize that a similar holistic, multidisciplinary approach should be recommended in skeletal dysplasias such as MO to improve the patient’s condition, focusing on comprehensive care that encompasses various aspects of the individual’s well-being.

Concerning the clinical presentation of MO patients, we found a significant association between the HRQoL and the number and location of OCs, deformities, and functional limitations, consistent with previous studies [18–20, 22]. In our study, the presence of one or more functional limitations, along with the deformities, increased the anxiety/depression in MO patients, compromising their ability to have a comfortable and fulfilling life. Other authors noticed that MO-induced limitations could restrict work ability and participation in sports, social, and recreational activities, thus amplifying a negative self-perception of QoL [18, 22]. This could compromise patients’ care as well as their compliance with proposed treatments. Therefore, we agree with the recommendations of Bathen et al., who emphasizes how crucial it is for healthcare professionals to listen to and understand the problems and needs of MO patients [21]. In this light, we recommend psychological assessment and support as part of a multidisciplinary approach to MO disease.

Caino et al. found an association between the IOR Classification and HRQoL [22]. Conversely, we could not demonstrate any relationship between the Classification and QoL, except for anxiety/depression dimension. There are several possible explanations for this finding. Despite the MO IOR Classification can identify the patient’s phenotype, it could not be adequate for describing the patients’ problems. For instance, a patient with restricted motion of a finger and partial loss of forearm supination is classified in a more severe class, compared with a patient with only one joint limitation, involving the hip. However, a painful limitation of the hip has a worse impact on the patient’s QoL compared to the reduced motion involving a forearm and a finger. Regrettably, the IOR Classification accounts only for the presence and number of limitations and not for the severity of each limitation itself. Second, the Classification is currently derived from medical charts, which could miss some information concerning the patient’s phenotype. Efforts should be made to develop a validated electronic disease-specific form for capturing and registering a thorough report of the patient’s phenotype, possibly scoring the severity of the various deformities and limitations. Third, we still lack a systematic radiographic screening program on a predefined schedule based on specific risk factors. This approach helps ensure that certain conditions or abnormalities are detected early. In our practice, MO patients undergo radiographs mainly after a physical examination, based on the symptoms they complained about. This aspect limits the reliability of the MO IOR Classification since the assigned class could be underrated in those patients who lack a thorough radiographic investigation.

This study reveals that MO concerns persist throughout life, and HRQoL tools can help assess their impact to ensure continuity and effective healthcare. Risk factors include increasing age, surgeries, and number of OCs, whereas young age, no surgeries, and a lower number of OCs are protective factors.

The study’s strength lies in capturing and combining the patient’s perspective with the physical examination, leading to a comprehensive understanding of the patient’s experiences at the time of the visit. Our results support the concept to create a dedicated multidisciplinary patient-centered healthcare pathway, including routine psychological assessment and PROMs administration for early identification of functional impairments, pain, and mood complaints, planning rehabilitation, intervention timing, and appropriateness of surgical procedures.

### Limitations of the study

This study has a few limitations. First, its cross-sectional design prevents the establishment of causality. Second, the study results are relevant to a single center. It is unclear whether they can be transferred to other centers. Lastly, the Spanish value set for EQ-5D-Y was applied to calculate the IV for children and adolescent participants. Although the EuroQol Group recommends using a value set comparable to the study population if the country-specific set is not yet available [42], it remains a shortcoming of our study since the lack of Italian young population norms restricted our comprehension of the disease’s impact on quality of life. To address these limitations, comprehensive longitudinal multicentric studies with large representative samples are warranted to accurately assess the overall disease-related burden of MO over time. Additionally, future research may consider other psychosocial factors that may influence HRQoL, such as educational attainment, occupational status, social connections/loneliness, and physical activity.

Despite these limitations, this study provides relevant information for HRQoL in MO patients and paves the way for further research into medical and non-medical therapeutic strategies for these patients.

### Supplementary Information

Below is the link to the electronic supplementary material.

Supplementary file1 (DOCX 28 kb)

Supplementary file2 (DOCX 17 kb)

Supplementary file3 (DOCX 16 kb)

Supplementary file4 (DOCX 16 kb)

Supplementary file5 (DOCX 76 kb)

Supplementary file6 (PPTX 137 kb)

## Data Availability

The de-identified dataset supporting the conclusions of this study can be made available from the corresponding author upon reasonable request, in agreement with EU and national legislation on the general data protection regulation.
