# Supplementary material for: Health-related quality of life and associated risk factors in patients with Multiple Osteochondromas: a cross-sectional study
Source: Qual Life Res. 2024 Mar 8;33(5):1323–34. doi: 10.1007/s11136-024-03604-4 (PMC11045590; doi:10.1007/s11136-024-03604-4)
Supplement: Supplementary file 2 — Supplementary file2 (DOCX 17 kb) [file 11136_2024_3604_MOESM2_ESM.docx]

**ST 1.** **Comparison of MO patients’ characteristics stratified by age group.**

| **Characteristics** | **Age Groups** | | | **p value** |
| --- | --- | --- | --- | --- |
|  | **2-7 years**  **(N=29)** | **8-15 years**  **(N=68)** | **≥16 years**  **(N=31)** |  |
| Age at visit, years | 6 (4, 7) | 12 (10, 14) | 25 (21, 34) | <0.001 |
| Comorbidity | 2 (7) | 9 (13) | 4 (13) | 0.66 |
| Height ^a^ |  |  |  |  |
| *cm* | 117.5 (106, 124.8) | 145.5 (131.5, 153) | 168 (161, 172) | <0.001 |
| *Z-score* | -0.1 (-0.7, 0.6) | -1.1 (-1.5, -0.1) | -0.7 (-1.2, -0.3) | 0.003 |
| Weight ^b^ |  |  |  |  |
| *kg* | 19.5 (17, 21.5) | 37 (30, 47) | 65 (53, 70) | <0.001 |
| *Z-score* | -0.3 (-0.7, 0.5) | -0.1 (-0.9, 0.2) | -0.1 (-0.7, 0.8) | 0.63 |
| BMI ^c^ |  |  |  |  |
| *kg/m^2^* | 14.9 (14.1, 15.6) | 18.3 (15.6, 20.6) | 22.6 (19.5, 24.8) | <0.001 |
| *Z-score* | -0.4 (-1.1, 0.1) | 0.1 (-0.6, 0.6) | 0.3 (-0.4, 0.8) | 0.18 |
| IOR Classification |  |  |  |  |
| *Class I* | 18 (62.1) | 7 (10.3) | 10 (32.3) | <0.001 |
| *Class II* | 6 (20.7) | 40 (58.8) | 12 (38.7) |  |
| *Class III* | 5 (17.2) | 21 (30.9) | 9 (29.0) |  |
| N. of OCs ^d^ | 7 (5, 9) | 11 (7, 16) | 10 (8, 16) | 0.001 |
| *Upper Limbs OCs* | 3 (1, 5) | 4 (2.5, 6) | 4 (2, 7) | 0.12 |
| *Lower Limbs OCs* | 3 (2, 5) | 6 (3, 9) | 7 (4, 8) | <0.001 |
| *Trunk OCs* | 0 (0, 2) | 0 (0, 1.5) | 1 (0, 2) | 0.82 |
| N. of Deformities | 0 (0, 2) | 3 (1, 4) | 1 (0, 3) | <0.001 |
| *Upper Limbs Deformities* | 0 (0, 0) | 1 (0, 2) | 0 (0, 1) | <0.001 |
| *Lower Limbs Deformities* | 0 (0, 0) | 2 (0, 2) | 0 (0, 2) | 0.002 |
| *Trunk Deformities* | 0 (0, 0) | 0 (0, 0) | 0 (0, 0) | 0.13 |
| N. of Limitations | 0 (0, 0) | 0 (0, 1) | 0 (0, 0) | 0.34 |
| *Upper Limbs Limitations* | 0 (0, 0) | 0 (0, 1) | 0 (0, 0) | 0.53 |
| *Lower Limbs Limitations* | 0 (0, 0) | 0 (0, 0) | 0 (0, 0) | 0.024 |
| *Trunk Limitations* | 0 (0, 0) | 0 (0, 0) | 0 (0, 0) | 0.21 |
| Age at first surgery, years | 4 (4, 5) | 9 (7, 10) | 13 (10, 21) | <0.001 |
| N. of Surgeries | 0 (0, 0) | 0 (0, 2) | 1 (0, 2) | <0.001 |
| Data are expressed as median and interquartile and *n* (%).  ^a^ = data were missing for 13 (10.2%) patients; ^b/c^ = data were missing for 17 (13.3%) patients.  ^d^ OCs: Osteochondromas. | | | | |
