# Supplementary material for: Health-related quality of life and associated risk factors in patients with Multiple Osteochondromas: a cross-sectional study
Source: Qual Life Res. 2024 Mar 8;33(5):1323–34. doi: 10.1007/s11136-024-03604-4 (PMC11045590; doi:10.1007/s11136-024-03604-4)
Supplement: Supplementary file 3 — Supplementary file3 (DOCX 16 kb) [file 11136_2024_3604_MOESM3_ESM.docx]

**ST 2. Comparison of MO patients’ characteristics stratified by surgery procedures.**

| **Characteristics** | **Surgery** | | **p value** |
| --- | --- | --- | --- |
|  | **No**  **(N=74)** | **Yes**  **(N=54)** |  |
| Age at visit, years | 9.5 (7, 13) | 14 (11, 20) | <0.001 |
| Comorbidity | 8 (10.8) | 7 (13.0) | 0.71 |
| Height ^a^ |  |  |  |
| *cm* | 136.8 (119, 151) | 153 (136, 163) | <0.001 |
| *Z-score* | -0.7 (-1.2, 0.3) | -0.8 (-1.5, 0.2) | 0.20 |
| Weight ^b^ |  |  |  |
| *kg* | 31 (21, 46) | 47 (34, 57) | <0.001 |
| *Z-score* | -0.2 (-0.7, 0.5) | -0.1 (-0.8, 0.3) | 1 |
| BMI ^c^ |  |  |  |
| *kg/m^2^* | 17.3 (14.9, 20.5) | 19.4 (15.6, 22) | 0.03 |
| *Z-score* | -0.1 (-0.8, 0.5) | 0.2 (-0.7, 0.7) | 0.40 |
| IOR Classification |  |  |  |
| *Class I* | 28 (37.8) | 7 (13.0) | 0.005 |
| *Class II* | 31 (41.9) | 27 (50.0) |  |
| *Class III* | 15 (20.3) | 20 (37.0) |  |
| N. of OCs ^d^ | 8 (6, 11) | 13 (10, 18) | <0.001 |
| *Upper Limbs OCs* | 3 (1, 5) | 4.5 (3, 8) | 0.004 |
| *Lower Limbs OCs* | 4 (3, 6) | 7 (4, 11) | <0.001 |
| *Trunk OCs* | 0 (0, 1) | 0 (0, 2) | 0.26 |
| N. of Deformities | 1 (0, 3) | 2 (1, 4) | 0.02 |
| *Upper Limbs Deformities* | 0 (0, 1) | 1 (0, 2) | 0.01 |
| *Lower Limbs Deformities* | 0 (0, 2) | 1 (0, 2) | 0.20 |
| *Trunk Deformities* | 0 (0, 0) | 0 (0, 0) | 0.18 |
| N. of Limitations | 0 (0, 0) | 0 (0, 1) | 0.04 |
| *Upper Limbs Limitations* | 0 (0, 0) | 0 (0, 1) | 0.01 |
| *Lower Limbs Limitations* | 0 (0, 0) | 0 (0, 0) | 0.66 |
| *Trunk Limitations* | 0 (0, 0) | 0 (0, 0) | 0.39 |
| Data are expressed as median and interquartile and *n* (%).  ^a^ = data were missing for 13 (10.2%) patients; ^b/c^ = data were missing for 17 (13.3%) patients.  ^d^ OCs: Osteochondromas. | | | |
