# Supplementary material for: Health-related quality of life and associated risk factors in patients with Multiple Osteochondromas: a cross-sectional study
Source: Qual Life Res. 2024 Mar 8;33(5):1323–34. doi: 10.1007/s11136-024-03604-4 (PMC11045590; doi:10.1007/s11136-024-03604-4)
Supplement: Supplementary file 4 — Supplementary file4 (DOCX 16 kb) [file 11136_2024_3604_MOESM4_ESM.docx]

**ST 3. Comparison of MO patients’ characteristics stratified by sex.**

| **Characteristics** | **Sex** | | **p value** |
| --- | --- | --- | --- |
|  | **Male**  **(N=61)** | **Female**  **(N=67)** |  |
| Age at visit, years | 12 (7, 16) | 12 (8, 15) | 0.68 |
| Comorbidity | 8 (13) | 7 (10) | 0.64 |
| Height ^a^ |  |  |  |
| *cm* | 147.5 (129.5, 168) | 145 (124, 156) | 0.061 |
| *Z-score* | -0.8 (-1.3, 0.2) | -0.7 (-1.4, 0) | 0.87 |
| Weight ^b^ |  |  |  |
| *kg* | 39 (26, 57) | 37 (24, 51) | 0.30 |
| *Z-score* | -0.6 (-0.8, 0.4) | 0 (-0.6, 0.5) | 0.23 |
| BMI ^c^ |  |  |  |
| *kg/m^2^* | 18.3 (15.3, 20.8) | 18.1 (15.2, 21) | 0.74 |
| *Z-score* | -0.1 (-0.6, 0.5) | 0.1 (-0.7, 0.7) | 0.32 |
| IOR Classification |  |  |  |
| *Class I* | 21 (34.4) | 14 (20.9) | 0.21 |
| *Class II* | 24 (39.3) | 34 (50.7) |  |
| *Class III* | 16 (26.2) | 19 (28.4) |  |
| N. of OCs ^d^ | 11 (7, 16) | 10 (7, 12) | 0.23 |
| *Upper Limbs OCs* | 4 (2, 7) | 4 (2, 5) | 0.58 |
| *Lower Limbs OCs* | 6 (3, 9) | 4 (3, 7) | 0.17 |
| *Trunk OCs* | 0 (0, 2) | 0 (0,1) | 0.17 |
| N. of Deformities | 1 (0, 3) | 2 (1, 4) | 0.099 |
| *Upper Limbs Deformities* | 0 (0, 1) | 0 (0, 2) | 0.57 |
| *Lower Limbs Deformities* | 0 (0, 2) | 1 (0, 2) | 0.22 |
| *Trunk Deformities* | 0 (0, 0) | 0 (0, 0) | 0.004 |
| N. of Limitations | 0 (0, 1) | 0 (0, 1) | 0.89 |
| *Upper Limbs Limitations* | 0 (0, 0) | 0 (0, 0) | 0.83 |
| *Lower Limbs Limitations* | 0 (0, 0) | 0 (0, 0) | 0.57 |
| *Trunk Limitations* | 0 (0, 0) | 0 (0, 0) | 0.34 |
| Age at first surgery, years | 8.5 (6, 11) | 10 (8, 12.5) | 0.11 |
| N. of Surgeries | 0 (0, 1) | 0 (0, 1) | 0.98 |
| Data are expressed as median and interquartile and *n* (%).  ^a^ = data were missing for 13 (10.2%) patients; ^b/c^ = data were missing for 17 (13.3%) patients.  ^d^ OCs: Osteochondromas. | | | |
