# Supplementary material for: Health-related quality of life and associated risk factors in patients with Multiple Osteochondromas: a cross-sectional study
Source: Qual Life Res. 2024 Mar 8;33(5):1323–34. doi: 10.1007/s11136-024-03604-4 (PMC11045590; doi:10.1007/s11136-024-03604-4)
Supplement: Supplementary file 5 — Supplementary file5 (DOCX 76 kb) [file 11136_2024_3604_MOESM5_ESM.docx]

**Supplementary Table 1. Multiple linear and logistic regression analyses on EQ-VAS and Five Dimensions (N=128)**

| **Characteristics** | **EQ-VAS** | | | | | | | | | | | | | | | | | | | | | | | | | | | |  |
| --- | --- | --- | --- | --- | --- | --- | --- | --- | --- | --- | --- | --- | --- | --- | --- | --- | --- | --- | --- | --- | --- | --- | --- | --- | --- | --- | --- | --- | --- |
|  | **Univariate regression** | | | | | |  | **Multiple regression** | | | | | | | | | | | | | | | | | | | | |  |
|  | **MD** | | **95% CI** | | **p** | |  | **Model 1** | | | | | |  | **Model 2** | | | | | |  | **Model 3** | | | | | | |  |
|  |  |  |  |  |  |  |  | **MD** | | | **95% CI** | | **p** |  | **MD** | | **95% CI** | | | **p** |  | **MD** | | | **95% CI** | | **p** | |  |
| Age at visit, *per* 1-year increase | -1.11 | | -1.38, -0.83 | | 0.000 | |  | -1.10 | | | -1.40, -0.80 | | 0.000 |  | -1.00 | | -1.29, -0.70 | | | 0.000 |  | -1.01 | | | -1.29, -0.72 | | 0.000 | |  |
| Comorbidity |  | |  | |  | |  |  | | |  | |  |  |  | |  | | |  |  |  | | |  | |  | |  |
| *Without (Ref.)* |  | | | |  | |  |  | | |  | |  |  |  | |  | | |  |  |  | | |  | |  | |  |
| *With* | -4.96 | | -15.44,5.52 | | 0.353 | |  | -1.65 | | | -10.17, 6.88 | | 0.703 |  | -3.61 | | -12.28, 5.07 | | | 0.412 |  | -4.51 | | | -12.94, 3.92 | | 0.292 | |  |
| BMI z-score, *per* 1-point increase | -1.66 | | -4.51,1.19 | | 0.252 | |  | -0.61 | | | -2.56, 1.33 | | 0.533 |  | -0.12 | | -2.07, 1.83 | | | 0.902 |  | -0.23 | | | -2.17, 1.71 | | 0.814 | |  |
| N. of OCs, *per* 1-OC increase | -0.40 | | -0.95, 0.16 | | 0.161 | |  |  | | |  | |  |  | 0.04 | | -0.53, 0.61 | | | 0.890 |  |  | | |  | |  | |  |
| *ULs OCs* | -0.01 | | -0.96, 0.94 | | 0.985 | |  | 0.58 | | | -0.28, 1.44 | | 0.186 |  |  | |  | | |  |  |  | | |  | |  | |  |
| *LLs OCs* | -0.78 | | -1.66, 0.10 | | 0.081 | |  | -0.14 | | | -0.97, 0.68 | | 0.731 |  |  | |  | | |  |  |  | | |  | |  | |  |
| *Trunk OCs* | -3.39 | | -6.90, 0.11 | | 0.058 | |  | -4.15 | | | -7.01, -1.29 | | 0.005 |  |  | |  | | |  |  |  | | |  | |  | |  |
| N. of Def, *per* 1-Def increase | -0.72 | | -2.11, 0.68 | | 0.313 | |  |  | | |  | |  |  | -0.11 | | -1.51, 1.29 | | | 0.873 |  |  | | |  | |  | |  |
| *ULs Def* | -2.20 | | -4.61, 0.21 | | 0.073 | |  | -1.28 | | | -3.67, 1.11 | | 0.290 |  |  | |  | | |  |  |  | | |  | |  | |  |
| *LLs Def* | 0.84 | | -1.23, 2.90 | | 0.428 | |  | 1.88 | | | 0.02, 3.74 | | 0.048 |  |  | |  | | |  |  |  | | |  | |  | |  |
| *Trunk Def* | -9.71 | | -16.71, -2.71 | | 0.007 | |  | -3.33 | | | -9.62, 2.96 | | 0.297 |  |  | |  | | |  |  |  | | |  | |  | |  |
| N. of Lim, *per* 1-Lim increase | -1.97 | | -5.50, 1.57 | | 0.276 | |  |  | | |  | |  |  | -0.04 | | -3.30, 3.21 | | | 0.980 |  |  | | |  | |  | |  |
| *ULs Lim* | -2.70 | | -7.64, 2.23 | | 0.283 | |  | -3.88 | | | -8.78, 1.01 | | 0.119 |  |  | |  | | |  |  |  | | |  | |  | |  |
| *LLs Lim* | -1.51 | | -7.02, 3.99 | | 0.590 | |  | 0.07 | | | -4.80, 4.94 | | 0.978 |  |  | |  | | |  |  |  | | |  | |  | |  |
| *Trunk Lim* | 6.00 | | -32.40, 44.40 | | 0.759 | |  | 38.72 | | | 2.00, 75.45 | | 0.039 |  |  | |  | | |  |  |  | | |  | |  | |  |
| IOR Class |  | |  | |  | |  |  | | |  | |  |  |  | |  | | |  |  |  | | |  | |  | |  |
| *Class I (Ref.)* |  | | | |  | |  |  | | |  | |  |  |  | |  | | |  |  |  | | |  | |  | |  |
| *Class II* | 1.10 | | -7.04, 9.25 | | 0.791 | |  |  | | |  | |  |  |  | |  | | |  |  | 6.20 | | | -0.69, 13.08 | | 0.077 | |  |
| *Class III* | -5.31 | | -14.41, 3.78 | | 0.252 | |  |  | | |  | |  |  |  | |  | | |  |  | 1.27 | | | -6.75, 9.29 | | 0.754 | |  |
| Surgery |  | |  | |  | |  |  | | |  | |  |  |  | |  | | |  |  |  | | |  | |  | |  |
| *Without (Ref.)* |  | | | |  | |  |  | | |  | |  |  |  | |  | | |  |  |  | | |  | |  | |  |
| *At least 1 surgery* | -12.99 | | -21.04, -4.94 | | 0.002 | |  | -5.08 | | | -12.85, 2.69 | | 0.198 |  | -7.87 | | -15.82, 0.08 | | | 0.052 |  | -8.30 | | | -15.90, -0.70 | | 0.033 | |  |
| *≥2 Surgeries* | -15.36 | | -23.41, -7.31 | | 0.000 | |  | -7.51 | | | -15.32, 0.30 | | 0.059 |  | -11.04 | | -18.96, -3.12 | | | 0.007 |  | -11.83 | | | -19.05, -4.61 | | 0.002 | |  |
| **Characteristics** | | **MOBILITY** | | | | | | | | | | | | | | | | | | | | | | | | | | |  |
|  |  | **Univariate regression** | | | | | | |  | **Multiple regression** | | | | | | | | | | | | | | | | | | |  |
|  |  | **OR^⸮^** | | **95% CI** | | **p** | | |  | **Model 1** | | | | | | |  | **Model 2** | | | | |  | **Model 3** | | | | |  |
|  |  |  |  |  |  |  |  |  |  | **OR** | | **95% CI** | | | | **p** |  | **OR** | **95% CI** | | **p** | |  | **OR** | | **95% CI** | | **p** |  |
| Age at visit, *per* 1-year increase | | 1.10 | | 1.05,1.15 | | 0.000 | | |  | 1.12 | | 1.06,1.19 | | | | 0.000 |  | 1.10 | 1.05,1.16 | | 0.000 | |  | 1.10 | | 1.05,1.16 | | 0.000 |  |
| Comorbidity | |  | |  | |  | | |  |  | |  | | | |  |  |  |  | |  | |  |  | |  | |  |  |
| *Without (Ref.)* | |  | | | |  | | |  |  | | | | | |  |  |  | | |  | |  |  | | | |  |  |
| *With* | | 0.88 | | 0.23,3.36 | | 0.852 | | |  | 1.06 | | 0.22,5.24 | | | | 0.941 |  | 0.75 | 0.15,3.71 | | 0.724 | |  | 0.76 | | 0.16,3.65 | | 0.734 |  |
| BMI z-score, *per* 1-point increase | | 0.99 | | 0.67,1.46 | | 0.945 | | |  | 1.07 | | 0.77,1.49 | | | | 0.667 |  | 1.08 | 0.80,1.47 | | 0.605 | |  | 1.16 | | 0.86,1.57 | | 0.338 |  |
| N. of OCs, *per* 1-OC increase | | 1.03 | | 0.96,1.10 | | 0.365 | | |  |  | |  | | | |  |  | 1.02 | 0.93,1.12 | | 0.723 | |  |  | |  | |  |  |
| *ULs OCs* | | 1.01 | | 0.90,1.13 | | 0.918 | | |  | 1.04 | | 0.88,1.23 | | | | 0.627 |  |  |  | |  | |  |  | |  | |  |  |
| *LLs OCs* | | 1.05 | | 0.95,1.17 | | 0.339 | | |  | 1.02 | | 0.88,1.18 | | | | 0.779 |  |  |  | |  | |  |  | |  | |  |  |
| *Trunk OCs* | | 1.39 | | 0.92,2.12 | | 0.121 | | |  | 1.52 | | 0.90,2.58 | | | | 0.120 |  |  |  | |  | |  |  | |  | |  |  |
| N. of Def, *per* 1-Def increase | | 1.07 | | 0.91,1.27 | | 0.394 | | |  |  | |  | | | |  |  | 1.03 | 0.81,1.30 | | 0.808 | |  |  | |  | |  |  |
| *ULs Def* | | 1.04 | | 0.77,1.39 | | 0.814 | | |  | 0.90 | | 0.58,1.41 | | | | 0.647 |  |  |  | |  | |  |  | |  | |  |  |
| *LLs Def* | | 1.13 | | 0.89,1.43 | | 0.305 | | |  | 1.02 | | 0.73,1.43 | | | | 0.906 |  |  |  | |  | |  |  | |  | |  |  |
| *Trunk Def* | | 0.83 | | 0.32,2.20 | | 0.712 | | |  | 0.46 | | 0.14,1.54 | | | | 0.207 |  |  |  | |  | |  |  | |  | |  |  |
| N. of Lim, *per* 1-Lim increase | | 1.24 | | 0.84,1.85 | | 0.282 | | |  |  | |  | | | |  |  | 1.00 | 0.61,1.64 | | 0.998 | |  |  | |  | |  |  |
| *ULs Lim* | | 0.93 | | 0.49,1.76 | | 0.814 | | |  | 1.14 | | 0.46,2.81 | | | | 0.776 |  |  |  | |  | |  |  | |  | |  |  |
| *LLs Lim* | | 1.74 | | 0.96,3.15 | | 0.066 | | |  | 1.37 | | 0.62,3.04 | | | | 0.437 |  |  |  | |  | |  |  | |  | |  |  |
| *Trunk Lim* | | 1.00 | | 1.00,1.00 | | . | | |  | 1.00 | | 1.00,1.00 | | | | . |  |  |  | |  | |  |  | |  | |  |  |
| IOR Class | |  | |  | |  | | |  |  | |  | | | |  |  |  |  | |  | |  |  | |  | |  |  |
| *Class I (Ref.)* | |  | | | |  | | |  |  | | | | | |  |  |  |  | |  | |  |  | | | |  |  |
| *Class II* | | 1.27 | | 0.46,3.54 | | 0.644 | | |  |  | |  | | | |  |  |  |  | |  | |  | 0.97 | | 0.27,3.43 | | 0.956 |  |
| *Class III* | | 1.00 | | 0.31,3.23 | | 1.000 | | |  |  | |  | | | |  |  |  |  | |  | |  | 0.54 | | 0.12,2.33 | | 0.405 |  |
| Surgery | |  | |  | |  | | |  |  | |  | | | |  |  |  |  | |  | |  |  | |  | |  |  |
| *Without (Ref.)* | |  | | | |  | | |  |  | | | | | |  |  |  | | |  | |  |  | | | |  |  |
| *At least 1 surgery* | | 2.86 | | 1.03,7.98 | | 0.044 | | |  | 1.70 | | 0.42,6.99 | | | | 0.460 |  | 2.16 | 0.61,7.63 | | 0.233 | |  | 2.75 | | 0.78,9.71 | | 0.116 |  |
| *≥2 Surgeries* | | 2.41 | | 0.85,6.86 | | 0.099 | | |  | 1.63 | | 0.42,6.41 | | | | 0.482 |  | 1.71 | 0.49,5.94 | | 0.399 | |  | 2.09 | | 0.63,6.86 | | 0.226 |  |
| **Characteristics** | | **SELF-CARE** | | | | | | | | | | | | | | | | | | | | | | | | | | |  |
|  |  | **Univariate regression** | | | | | | |  | **Multiple regression** | | | | | | | | | | | | | | | | | | |  |
|  |  | **OR** | | **95% CI** | | **p** | | |  | **Model 1** | | | | | | |  | **Model 2** | | | | |  | **Model 3** | | | | |  |
|  |  |  |  |  |  |  |  |  |  | **OR** | | **95% CI** | | | | **p** |  | **OR** | **95% CI** | | **p** | |  | **OR** | | **95% CI** | | **p** |  |
| Age at visit, *per* 1-year increase | | 1.10 | | 1.05, 1.16 | | 0.000 | | |  | 1.18 | | 1.08, 1.29 | | | | 0.000 |  | 1.14 | 1.06, 1.23 | | 0.001 | |  | 1.13 | | 1.06, 1.21 | | 0.000 |  |
| Comorbidity | |  | |  | |  | | |  |  | |  | | | |  |  |  |  | |  | |  |  | |  | |  |  |
| *Without (Ref.)* | |  | | | |  | | |  |  | |  | | | |  |  |  |  | |  | |  |  | |  | |  |  |
| *With* | | 3.75 | | 1.00, 13.96 | | 0.049 | | |  | 12.04 | | 1.00, 144.82 | | | | 0.050 |  | 9.86 | 1.42, 68.58 | | 0.021 | |  | 7.99 | | 1.30, 49.06 | | 0.025 |  |
| BMI z-score, *per* 1-point increase | | 0.94 | | 0.56, 1.59 | | 0.823 | | |  | 0.91 | | 0.55, 1.49 | | | | 0.702 |  | 0.98 | 0.64, 1.49 | | 0.924 | |  | 1.06 | | 0.71, 1.59 | | 0.781 |  |
| N. of OCs, *per* 1-OC increase | | 1.04 | | 0.95, 1.13 | | 0.431 | | |  |  | |  | | | |  |  | 0.95 | 0.82, 1.11 | | 0.548 | |  |  | |  | |  |  |
| *ULs OCs* | | 0.96 | | 0.80, 1.14 | | 0.625 | | |  | 0.79 | | 0.53, 1.18 | | | | 0.252 |  |  |  | |  | |  |  | |  | |  |  |
| *LLs OCs* | | 1.07 | | 0.93, 1.23 | | 0.317 | | |  | 1.11 | | 0.85, 1.44 | | | | 0.458 |  |  |  | |  | |  |  | |  | |  |  |
| *Trunk OCs* | | 2.07 | | 1.21, 3.57 | | 0.008 | | |  | 4.10 | | 1.34, 12.54 | | | | 0.013 |  |  |  | |  | |  |  | |  | |  |  |
| N. of Def, *per* 1-Def increase | | 1.28 | | 1.05, 1.56 | | 0.016 | | |  |  | |  | | | |  |  | 1.39 | 0.97, 1.99 | | 0.072 | |  |  | |  | |  |  |
| *ULs Def* | | 1.47 | | 1.05, 2.06 | | 0.023 | | |  | 1.00 | | 0.49, 2.07 | | | | 0.990 |  |  |  | |  | |  |  | |  | |  |  |
| *LLs Def* | | 1.19 | | 0.91, 1.57 | | 0.208 | | |  | 1.19 | | 0.70, 2.02 | | | | 0.519 |  |  |  | |  | |  |  | |  | |  |  |
| *Trunk Def* | | 1.95 | | 0.80, 4.74 | | 0.141 | | |  | 3.64 | | 0.33, 39.78 | | | | 0.289 |  |  |  | |  | |  |  | |  | |  |  |
| N. of Lim, *per* 1-Lim increase | | 1.50 | | 0.96, 2.35 | | 0.072 | | |  |  | |  | | | |  |  | 1.22 | 0.65, 2.31 | | 0.538 | |  |  | |  | |  |  |
| *ULs Lim* | | 1.27 | | 0.63, 2.54 | | 0.504 | | |  | 3.44 | | 0.69, 17.30 | | | | 0.133 |  |  |  | |  | |  |  | |  | |  |  |
| *LLs Lim* | | 1.87 | | 1.01, 3.46 | | 0.046 | | |  | 1.72 | | 0.60, 4.95 | | | | 0.316 |  |  |  | |  | |  |  | |  | |  |  |
| *Trunk Lim* | | 1.00 | | 1.00, 1.00 | | . | | |  | 1.00 | | 1.00, 1.00 | | | | . |  |  |  | |  | |  |  | |  | |  |  |
| IOR Class | |  | |  | |  | | |  |  | |  | | | |  |  |  |  | |  | |  |  | |  | |  |  |
| *Class I (Ref.)* | |  | | | |  | | |  |  | |  | | | |  |  |  |  | |  | |  |  | |  | |  |  |
| *Class II* | | 1.90 | | 0.36, 10.00 | | 0.447 | | |  |  | |  | | | |  |  |  |  | |  | |  | 1.64 | | 0.21, 12.65 | | 0.638 |  |
| *Class III* | | 3.41 | | 0.64, 18.25 | | 0.151 | | |  |  | |  | | | |  |  |  |  | |  | |  | 3.70 | | 0.46, 30.05 | | 0.221 |  |
| Surgery | |  | |  | |  | | |  |  | |  | | | |  |  |  |  | |  | |  |  | |  | |  |  |
| *Without (Ref.)* | |  | | | |  | | |  |  | |  | | | |  |  |  |  | |  | |  |  | |  | |  |  |
| *At least 1 surgery* | | 6.13 | | 1.63, 23.05 | | 0.007 | | |  | 3.05 | | 0.30, 30.71 | | | | 0.343 |  | 6.04 | 0.95, 38.30 | | 0.056 | |  | 6.50 | | 1.12, 37.80 | | 0.037 |  |
| *≥2 Surgeries* | | 2.19 | | 0.46, 10.48 | | 0.328 | | |  | 0.69 | | 0.05, 8.73 | | | | 0.772 |  | 1.81 | 0.24, 13.68 | | 0.567 | |  | 1.60 | | 0.26, 9.73 | | 0.611 |  |
| **Characteristics** | | **USUAL ACTIVITIES** | | | | | | | | | | | | | | | | | | | | | | | | | | |  |
|  |  | **Univariate regression** | | | | | | |  | **Multiple regression** | | | | | | | | | | | | | | | | | | |  |
|  |  | **OR** | | **95% CI** | | **p** | | |  | **Model 1** | | | | | | |  | **Model 2** | | | | |  | **Model 3** | | | | |  |
|  |  |  |  |  |  |  |  |  |  | **OR** | | **95% CI** | | | | **p** |  | **OR** | **95% CI** | | **p** | |  | **OR** | | **95% CI** | | **p** |  |
| Age at visit, *per* 1-year increase | | 1.14 | | 1.08, 1.21 | | 0.000 | | |  | 1.15 | | 1.08, 1.23 | | | | 0.000 |  | 1.14 | 1.07, 1.21 | | 0.000 | |  | 1.14 | | 1.07, 1.21 | | 0.000 |  |
| Comorbidity | |  | |  | |  | | |  |  | |  | | | |  |  |  |  | |  | |  |  | |  | |  |  |
| *Without (Ref.)* | |  | | | |  | | |  |  | |  | | | |  |  |  |  | |  | |  |  | |  | |  |  |
| *With* | | 1.16 | | 0.34, 3.94 | | 0.814 | | |  | 0.61 | | 0.11, 3.41 | | | | 0.577 |  | 1.13 | 0.25, 5.15 | | 0.875 | |  | 1.15 | | 0.26, 5.02 | | 0.853 |  |
| BMI z-score, *per* 1-point increase | | 1.05 | | 0.72, 1.53 | | 0.812 | | |  | 0.89 | | 0.65, 1.21 | | | | 0.447 |  | 0.93 | 0.70, 1.25 | | 0.643 | |  | 1.01 | | 0.75, 1.34 | | 0.963 |  |
| N. of OCs, *per* 1-OC increase | | 1.06 | | 0.99, 1.13 | | 0.085 | | |  |  | |  | | | |  |  | 1.09 | 0.99, 1.21 | | 0.081 | |  |  | |  | |  |  |
| *ULs OCs* | | 0.98 | | 0.88, 1.11 | | 0.800 | | |  | 0.93 | | 0.78, 1.11 | | | | 0.436 |  |  |  | |  | |  |  | |  | |  |  |
| *LLs OCs* | | 1.16 | | 1.04, 1.29 | | 0.007 | | |  | 1.25 | | 1.06, 1.46 | | | | 0.006 |  |  |  | |  | |  |  | |  | |  |  |
| *Trunk OCs* | | 1.24 | | 0.82, 1.87 | | 0.306 | | |  | 1.57 | | 0.92, 2.68 | | | | 0.099 |  |  |  | |  | |  |  | |  | |  |  |
| N. of Def, *per* 1-Def increase | | 1.03 | | 0.88, 1.22 | | 0.683 | | |  |  | |  | | | |  |  | 0.92 | 0.71,1.20 | | 0.545 | |  |  | |  | |  |  |
| *ULs Def* | | 1.15 | | 0.87, 1.51 | | 0.323 | | |  | 1.35 | | 0.86, 2.13 | | | | 0.196 |  |  |  | |  | |  |  | |  | |  |  |
| *LLs Def* | | 0.92 | | 0.70, 1.21 | | 0.565 | | |  | 0.75 | | 0.46, 1.21 | | | | 0.240 |  |  |  | |  | |  |  | |  | |  |  |
| *Trunk Def* | | 1.57 | | 0.72, 3.42 | | 0.254 | | |  | 0.75 | | 0.22, 2.59 | | | | 0.651 |  |  |  | |  | |  |  | |  | |  |  |
| N. of Lim, *per* 1-Lim increase | | 1.31 | | 0.89, 1.92 | | 0.173 | | |  |  | |  | | | |  |  | 1.14 | 0.65, 1.99 | | 0.638 | |  |  | |  | |  |  |
| *ULs Lim* | | 1.31 | | 0.76, 2.26 | | 0.324 | | |  | 1.06 | | 0.41, 2.76 | | | | 0.898 |  |  |  | |  | |  |  | |  | |  |  |
| *LLs Lim* | | 1.27 | | 0.70, 2.28 | | 0.429 | | |  | 1.00 | | 0.37, 2.67 | | | | 1.000 |  |  |  | |  | |  |  | |  | |  |  |
| *Trunk Lim* | | 1.00 | | 1.00, 1.00 | | . | | |  | 1.00 | | 1.00, 1.00 | | | | . |  |  |  | |  | |  |  | |  | |  |  |
| IOR Class | |  | |  | |  | | |  |  | |  | | | |  |  |  |  | |  | |  |  | |  | |  |  |
| *Class I (Ref.)* | |  | | | |  | | |  |  | |  | | | |  |  |  |  | |  | |  |  | |  | |  |  |
| *Class II* | | 1.07 | | 0.40, 2.90 | | 0.888 | | |  |  | |  | | | |  |  |  |  | |  | |  | 0.87 | | 0.24, 3.22 | | 0.836 |  |
| *Class III* | | 1.17 | | 0.39, 3.49 | | 0.781 | | |  |  | |  | | | |  |  |  |  | |  | |  | 0.75 | | 0.17, 3.23 | | 0.695 |  |
| Surgery | |  | |  | |  | | |  |  | |  | | | |  |  |  |  | |  | |  |  | |  | |  |  |
| *Without (Ref.)* | |  | | | |  | | |  |  | |  | | | |  |  |  |  | |  | |  |  | |  | |  |  |
| *At least 1 surgery* | | 1.98 | | 0.71, 5.48 | | 0.191 | | |  | 0.50 | | 0.11, 2.26 | | | | 0.368 |  | 0.80 | 0.20, 3.21 | | 0.749 | |  | 1.23 | | 0.32, 4.69 | | 0.759 |  |
| *≥2 Surgeries* | | 2.76 | | 1.03, 7.39 | | 0.043 | | |  | 1.22 | | 0.30, 4.95 | | | | 0.780 |  | 1.33 | 0.37, 4.72 | | 0.659 | |  | 2.27 | | 0.71, 7.29 | | 0.167 |  |
| **Characteristics** | | **PAIN/DISCOMFORT** | | | | | | | | | | | | | | | | | | | | | | | | | | |  |
|  |  | **Univariate regression** | | | | | | |  | **Multiple regression** | | | | | | | | | | | | | | | | | | |  |
|  |  | **OR** | | **95% CI** | | **p** | | |  | **Model 1** | | | | | | |  | **Model 2** | | | | |  | **Model 3** | | | | |  |
|  |  |  |  |  |  |  |  |  |  | **OR** | | **95% CI** | | | | **p** |  | **OR** | **95% CI** | | **p** | |  | **OR** | | **95% CI** | | **p** |  |
| Age at visit, *per* 1-year increase | | 1.10 | | 1.04, 1.16 | | 0.001 | | |  | 1.13 | | 1.05, 1.20 | | | | 0.000 |  | 1.09 | 1.03, 1.15 | | 0.002 | |  | 1.09 | | 1.03, 1.15 | | 0.002 |  |
| Comorbidity | |  | |  | |  | | |  |  | |  | | | |  |  |  |  | |  | |  |  | |  | |  |  |
| *Without (Ref.)* | |  | | | |  | | |  |  | |  | | | |  |  |  |  | |  | |  |  | |  | |  |  |
| *With* | | 1.64 | | 0.55, 4.91 | | 0.377 | | |  | 1.68 | | 0.46, 6.18 | | | | 0.433 |  | 1.63 | 0.50, 5.33 | | 0.419 | |  | 1.69 | | 0.52, 5.46 | | 0.383 |  |
| BMI z-score, *per* 1-point increase | | 1.11 | | 0.80, 1.53 | | 0.532 | | |  | 1.07 | | 0.81, 1.43 | | | | 0.626 |  | 0.98 | 0.77, 1.27 | | 0.906 | |  | 1.00 | | 0.78, 1.28 | | 0.981 |  |
| N. of OCs, *per* 1-OC increase | | 1.06 | | 0.99, 1.12 | | 0.079 | | |  |  | |  | | | |  |  | 1.03 | 0.96, 1.12 | | 0.398 | |  |  | |  | |  |  |
| *ULs OCs* | | 1.08 | | 0.97, 1.21 | | 0.136 | | |  | 1.10 | | 0.96, 1.25 | | | | 0.163 |  |  |  | |  | |  |  | |  | |  |  |
| *LLs OCs* | | 1.03 | | 0.94, 1.13 | | 0.503 | | |  | 0.96 | | 0.85, 1.09 | | | | 0.577 |  |  |  | |  | |  |  | |  | |  |  |
| *Trunk OCs* | | 1.78 | | 1.20, 2.65 | | 0.005 | | |  | 2.02 | | 1.26, 3.24 | | | | 0.003 |  |  |  | |  | |  |  | |  | |  |  |
| N. of Def, *per* 1-Def increase | | 1.07 | | 0.93, 1.24 | | 0.334 | | |  |  | |  | | | |  |  | 1.01 | 0.83, 1.23 | | 0.893 | |  |  | |  | |  |  |
| *ULs Def* | | 1.21 | | 0.93, 1.57 | | 0.150 | | |  | 1.07 | | 0.73, 1.55 | | | | 0.734 |  |  |  | |  | |  |  | |  | |  |  |
| *LLs Def* | | 1.03 | | 0.83, 1.28 | | 0.772 | | |  | 0.87 | | 0.65, 1.16 | | | | 0.337 |  |  |  | |  | |  |  | |  | |  |  |
| *Trunk Def* | | 1.02 | | 0.49, 2.14 | | 0.948 | | |  | 0.52 | | 0.18, 1.56 | | | | 0.245 |  |  |  | |  | |  |  | |  | |  |  |
| N. of Lim, *per* 1-Lim increase | | 1.21 | | 0.83, 1.77 | | 0.316 | | |  |  | |  | | | |  |  | 1.01 | 0.64, 1.60 | | 0.964 | |  |  | |  | |  |  |
| *ULs Lim* | | 1.02 | | 0.61, 1.70 | | 0.935 | | |  | 1.30 | | 0.63, 2.68 | | | | 0.482 |  |  |  | |  | |  |  | |  | |  |  |
| *LLs Lim* | | 1.74 | | 0.84, 3.62 | | 0.137 | | |  | 1.47 | | 0.65, 3.30 | | | | 0.353 |  |  |  | |  | |  |  | |  | |  |  |
| *Trunk Lim* | | 1.00 | | 1.00,1.00 | | . | | |  | 1.00 | | 1.00, 1.00 | | | | . |  |  |  | |  | |  |  | |  | |  |  |
| IOR Class | |  | |  | |  | | |  |  | |  | | | |  |  |  |  | |  | |  |  | |  | |  |  |
| *Class I (Ref.)* | |  | | | |  | | |  |  | |  | | | |  |  |  |  | |  | |  |  | |  | |  |  |
| *Class II* | | 1.61 | | 0.69, 3.76 | | 0.274 | | |  |  | |  | | | |  |  |  |  | |  | |  | 1.30 | | 0.49, 3.46 | | 0.601 |  |
| *Class III* | | 1.78 | | 0.69, 4.60 | | 0.233 | | |  |  | |  | | | |  |  |  |  | |  | |  | 1.27 | | 0.41, 3.92 | | 0.676 |  |
| Surgery | |  | |  | |  | | |  |  | |  | | | |  |  |  |  | |  | |  |  | |  | |  |  |
| *Without (Ref.)* | |  | | | |  | | |  |  | |  | | | |  |  |  |  | |  | |  |  | |  | |  |  |
| *At least 1 surgery* | | 2.13 | | 0.87, 5.23 | | 0.098 | | |  | 1.07 | | 0.31, 3.71 | | | | 0.912 |  | 1.32 | 0.46, 3.81 | | 0.607 | |  | 1.50 | | 0.54, 4.17 | | 0.439 |  |
| *≥2 Surgeries* | | 2.49 | | 1.01, 6.19 | | 0.049 | | |  | 1.51 | | 0.45, 5.09 | | | | 0.504 |  | 1.46 | 0.51, 4.20 | | 0.483 | |  | 1.68 | | 0.62, 4.56 | | 0.311 |  |
| **Characteristics** | | **ANXIETY/DEPRESSION** | | | | | | | | | | | | | | | | | | | | | | | | | | |  |
|  |  | **Univariate regression** | | | | | | |  | **Multiple regression** | | | | | | | | | | | | | | | | | | |  |
|  |  | **OR** | | **95% CI** | | **p** | | |  | **Model 1** | | | | | | |  | **Model 2** | | | | |  | **Model 3** | | | | |  |
|  |  |  |  |  |  |  |  |  |  | **OR** | | **95% CI** | | | | **p** |  | **OR** | **95% CI** | | **p** | |  | **OR** | | **95% CI** | | **p** |  |
| Age at visit, *per* 1-year increase | | 1.08 | | 1.03, 1.13 | | 0.001 | | |  | 1.07 | | 1.02, 1.13 | | | | 0.007 |  | 1.08 | 1.03, 1.13 | | 0.002 | |  | 1.08 | | 1.03, 1.13 | | 0.001 |  |
| Comorbidity | |  | |  | |  | | |  |  | |  | | | |  |  |  |  | |  | |  |  | |  | |  |  |
| *Without (Ref.)* | |  | | | |  | | |  |  | |  | | | |  |  |  |  | |  | |  |  | |  | |  |  |
| *With* | | 2.12 | | 0.71, 6.32 | | 0.177 | | |  | 1.92 | | 0.50, 7.35 | | | | 0.341 |  | 2.46 | 0.71, 8.44 | | 0.154 | |  | 2.49 | | 0.72, 8.67 | | 0.152 |  |
| BMI z-score, *per* 1-point increase | | 1.07 | | 0.76, 1.51 | | 0.712 | | |  | 1.14 | | 0.84,1.55 | | | | 0.390 |  | 1.15 | 0.86, 1.53 | | 0.345 | |  | 1.14 | | 0.86, 1.51 | | 0.348 |  |
| N. of OCs, *per* 1-OC increase | | 1.06 | | 0.99, 1.12 | | 0.074 | | |  |  | |  | | | |  |  | 1.00 | 0.92, 1.09 | | 0.940 | |  |  | |  | |  |  |
| *ULs OCs* | | 1.00 | | 0.90, 1.11 | | 0.987 | | |  | 0.90 | | 0.77, 1.04 | | | | 0.146 |  |  |  | |  | |  |  | |  | |  |  |
| *LLs OCs* | | 1.14 | | 1.03, 1.26 | | 0.009 | | |  | 1.06 | | 0.93, 1.21 | | | | 0.355 |  |  |  | |  | |  |  | |  | |  |  |
| *Trunk OCs* | | 1.17 | | 0.80, 1.72 | | 0.424 | | |  | 1.25 | | 0.79, 1.99 | | | | 0.338 |  |  |  | |  | |  |  | |  | |  |  |
| N. of Def, *per* 1-Def increase | | 1.14 | | 0.98, 1.33 | | 0.087 | | |  |  | |  | | | |  |  | 1.02 | 0.82, 1.27 | | 0.844 | |  |  | |  | |  |  |
| *ULs Def* | | 1.29 | | 0.99, 1.67 | | 0.057 | | |  | 1.20 | | 0.82, 1.77 | | | | 0.352 |  |  |  | |  | |  |  | |  | |  |  |
| *LLs Def* | | 1.02 | | 0.82, 1.28 | | 0.832 | | |  | 0.86 | | 0.59, 1.24 | | | | 0.406 |  |  |  | |  | |  |  | |  | |  |  |
| *Trunk Def* | | 2.21 | | 0.99, 4.93 | | 0.053 | | |  | 2.15 | | 0.69, 6.67 | | | | 0.185 |  |  |  | |  | |  |  | |  | |  |  |
| N. of Lim, *per* 1-Lim increase | | 1.59 | | 1.07, 2.37 | | 0.023 | | |  |  | |  | | | |  |  | 1.38 | 0.85, 2.22 | | 0.193 | |  |  | |  | |  |  |
| *ULs Lim* | | 1.49 | | 0.88, 2.53 | | 0.138 | | |  | 1.18 | | 0.55, 2.53 | | | | 0.677 |  |  |  | |  | |  |  | |  | |  |  |
| *LLs Lim* | | 1.71 | | 0.93, 3.16 | | 0.085 | | |  | 1.59 | | 0.75, 3.37 | | | | 0.227 |  |  |  | |  | |  |  | |  | |  |  |
| *Trunk Lim* | | 1.00 | | 1.00, 1.00 | | . | | |  | 1.00 | | 1.00, 1.00 | | | | . |  |  |  | |  | |  |  | |  | |  |  |
| IOR Class | |  | |  | |  | | |  |  | |  | | | |  |  |  |  | |  | |  |  | |  | |  |  |
| *Class I (Ref.)* | |  | | | |  | | |  |  | |  | | | |  |  |  |  | |  | |  |  | |  | |  |  |
| *Class II* | | 2.17 | | 0.77, 6.16 | | 0.143 | | |  |  | |  | | | |  |  |  |  | |  | |  | 1.62 | | 0.48, 5.49 | | 0.436 |  |
| *Class III* | | 4.07 | | 1.35, 12.26 | | 0.013 | | |  |  | |  | | | |  |  |  |  | |  | |  | 2.77 | | 0.75, 10.24 | | 0.126 |  |
| Surgery | |  | |  | |  | | |  |  | |  | | | |  |  |  |  | |  | |  |  | |  | |  |  |
| *Without (Ref.)* | |  | | | |  | | |  |  | |  | | | |  |  |  |  | |  | |  |  | |  | |  |  |
| *At least 1 surgery* | | 3.15 | | 1.22, 8.11 | | 0.018 | | |  | 2.75 | | 0.80, 9.47 | | | | 0.109 |  | 2.71 | 0.86, 8.50 | | 0.089 | |  | 2.59 | | 0.85, 7.87 | | 0.093 |  |
| *≥2 Surgeries* | | 3.65 | | 1.42, 9.39 | | 0.007 | | |  | 2.52 | | 0.76, 8.32 | | | | 0.131 |  | 2.68 | 0.88, 8.19 | | 0.084 | |  | 2.36 | | 0.83, 6.68 | | 0.105 |  |
| a Model 1: Adjusted by all the variables in the Model 1  b Model 2: Adjusted by all the variables in the Model 2  c Model 3: Adjusted by all the variables in the Model 3  **^⸙^** MD: Mean Difference; **^±^** CI: Confidence Interval; ^⸸^ OCs: Osteochondromas; ^⸿^ ULs: Upper Limbs; ¥ LLs: Lower Limbs; ^~^ Def: Deformities; ^§^ Lim: Limitations | | | | | | | | | | | | | | | | | | | | | | | | | | | | |  |
| **^⸮^** OR: Odds Ratio | | | | | | | | | | | | | | | | | | | | | | | | | | | | |  |
