# Supplementary material for: Health-related quality of life and associated risk factors in patients with Multiple Osteochondromas: a cross-sectional study
Source: Qual Life Res. 2024 Mar 8;33(5):1323–34. doi: 10.1007/s11136-024-03604-4 (PMC11045590; doi:10.1007/s11136-024-03604-4)
Supplement: Supplementary file 6 — Supplementary file6 (PPTX 137 kb) [file 11136_2024_3604_MOESM6_ESM.pptx]

## Slide 1
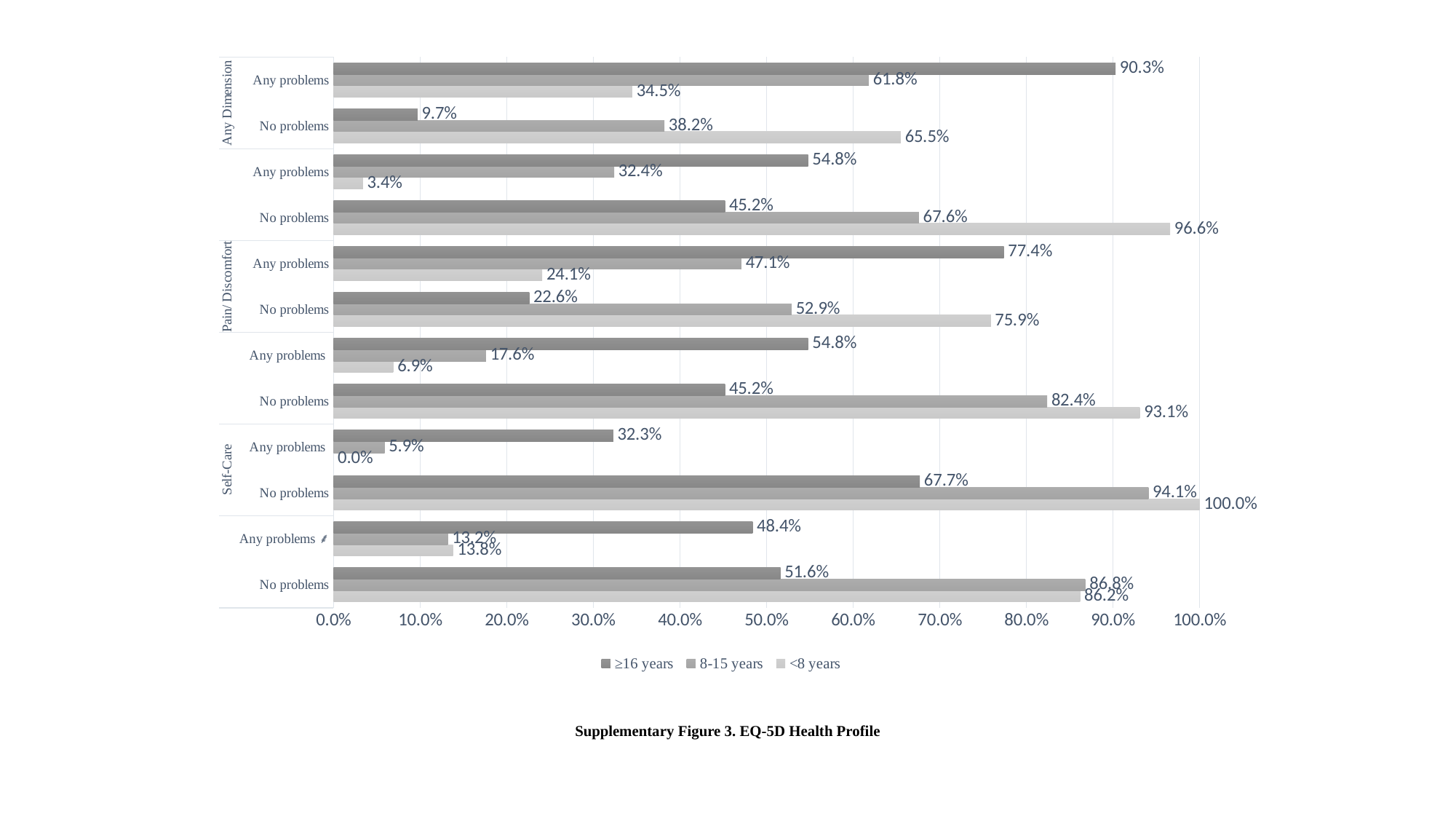

### Chart
| Category | <8 years | 8-15 years | ≥16 years |
|---|---|---|---|
| No problems | 0.862 | 0.868 | 0.516 |
| Any problems ⸙ | 0.138 | 0.132 | 0.484 |
| No problems | 1.0 | 0.941 | 0.677 |
| Any problems | 0.0 | 0.059 | 0.323 |
| No problems | 0.931 | 0.824 | 0.452 |
| Any problems | 0.069 | 0.176 | 0.548 |
| No problems | 0.759 | 0.529 | 0.226 |
| Any problems | 0.241 | 0.471 | 0.774 |
| No problems | 0.966 | 0.676 | 0.452 |
| Any problems | 0.034 | 0.324 | 0.548 |
| No problems | 0.655 | 0.382 | 0.097 |
| Any problems | 0.345 | 0.618 | 0.903 |Supplementary Figure 3. EQ-5D Health Profile
